# A cross-sectional exploratory study of food literacy among Saudi parents of adolescent children aged 10 to 19 years

**DOI:** 10.3389/fnut.2022.1083118

**Published:** 2023-01-06

**Authors:** Khlood Bookari

**Affiliations:** ^1^Department of Clinical Nutrition, Faculty of Applied Medical Sciences, Taibah University, Medina, Saudi Arabia; ^2^National Nutrition Committee (NNC), Saudi Food and Drug Authority (SFDA), Riyadh, Saudi Arabia

**Keywords:** parental, food literacy, Saudi, parents, correlates

## Abstract

**Introduction:**

Parental food literacy is fundamental in laying a solid foundation for healthy eating among their children. This study aimed to (1) evaluate the current state of food literacy among Saudi parents of 10-19 years old adolescent children, and (2) determine the correlates associated with parental food literacy.

**Methods:**

This cross-sectional study was conducted between April and June 2022, with a convenience sample of 1845 Saudi parents (mean age = 45.1 ± 11; mothers: 56%). A Short Food Literacy Questionnaire (SFLQ) was used to meet the study aims.

**Results:**

Findings showed that around 46% of parents had poor food literacy. Fathers were 2.4 times more likely than mothers to be food illiterate (OR = 2.4, CI = 1.9–3.0, *p* < 0.001). Parents residing in Riyadh, Northern borders, Jawf, or Ha’il had a three times higher risk of being food illiterate than those residing in other provinces (OR = 3.2, CI = 2.6–3.9, *p* < 0.001). Parental overweight or obesity increased their risk of being food illiterate by 60% (OR = 1.6, CI = 1.3–2.1, *p* < 0.001). Healthy parents, in contrast to those having a chronic disease (s), had a 60% higher probability of food illiteracy (OR = 0.4, CI = 0.3–0.6, *p* < 0.001). Educated parents had a three times higher risk of being food illiterate (vs. uneducated parents, OR = 3.0, CI = 1.6–5.8, *p* = 0.001). Parents making less than 3000 Saudi Riyal (SR) per month (<798 USD/779 EUR) were 40% more likely to be food illiterate than those who reported 3000-25000 SR (798–6652 USD/779–6499 EUR) (OR = 0.6, CI = 0.4–0.9, *p* = 0.02), and 70% more likely to be food illiterate than those making more than 25000 SR (>6652 USD/6497 EUR) (OR = 0.3, CI = 0.2–0.6, *p* < 0.001). Parents who lived in crowded households were twice as likely to be food illiterate as those who did not (OR = 1.9, CI = 1.5–2.4, *p* < 0.001).

**Conclusion:**

The current study findings should be employed in future programming and policy-making approaches to reach Saudi parents for necessary food literacy interventions. These interventions could include bolstering their confidence while cooking, buying groceries, reading nutrition labels, and integrating them into nutrition education lessons with their children in school settings.

## 1. Introduction

Food literacy is a construct that affects an individual’s ability to comprehend food and nutrition information, understand food labels, practice food safety precautions, cook healthily and safely, and adhere to dietary recommendations in their food choices (1, 2). A food-literate person makes decisions to support the achievement of personal health and a sustainable food system considering environmental, social, economic, cultural, and political components (3). It is “the scaffolding term that empowers individuals, households, communities, or nations to protect diet quality through change and strengthen dietary resilience over time” (4). In the early 90s, food literacy was first defined to exemplify a person’s functional ability to maintain a nutritious diet without being deprived (5). Since then, food literacy topics have evolved over the years and have caught the interest of nutrition researchers (6). In the Middle East and North Africa (MENA) region, individuals’ food literacy predicted their food habits, food label use, food consumption patterns, school performance, dietary diversity, nutrient adequacy, and food security (6). According to the Food and Agricultural Organization (FAO), nearly one-third of Arabs were food insecure in 2020 (7). Additionally, between 2014 and 2020, the number of undernourished people in the Arab world increased by 30%, reaching 69 million (7). By 2030, there will likely be more than 75 million undernourished Arab people (7). The prevalence of obesity increased concurrently to 28.8% in 2020, more than double the global average of 13.1%, placing the Arab region as the third most obese in the world (7).

Saudi Arabia has progressed somewhat toward its diet-related goals for preventing and controlling non-communicable diseases (NCD) (8). In accordance with the 2025 global nutrition goals, Saudi Arabia attempted to prevent a rise in childhood obesity, and its efforts to prevent noncommunicable diseases centered on limiting people’s exposure to modifiable risk factors (8). With an estimated 34.3% of adult men and 45.5% of adult women living with obesity, the country has made little progress toward its obesity prevention goal (8). During the upcoming years, it is anticipated that the prevalence of adolescent obesity in Saudi Arabia will continue to rise. The latter claim is backed by frequent observations of Saudi adolescents’ fast-food consumption, skipping breakfast, and minimal consumption of fruits and vegetables (9).

After infancy, the adolescent years (about 10–19) provide a second, equally important opportunity to foster healthy growth and maturation (10). The fast physical growth during adolescence is known as the pubertal growth spurt (11), accounting for about 20–25% of the final adult height (11). Moreover, adolescents potentially gain 50% of their ideal body weight in this growth spurt (11). Positive environments and relations can improve developmental outcomes during this time, while the effects of negative experiences can be amplified and last into adulthood (10). Parents are crucial in determining how teenagers respond to the various elements that define their development (12). As children progress into adolescence, the parenting relationship changes, and parents need different developmentally-appropriate skills and tactics to meet the needs of their children (12). Adequate parental support improves adolescents’ developmental outcomes and can mitigate the influence of unfavorable external factors (12). Additionally, the impact of parenting practices might outlast generations (12). Parenting programs are, broadly, “a combination of activities or services designed to improve how parents approach and carry out their parental role specifically their parenting knowledge, attitudes, skills, and practices” (10).

Parents can influence their children’s evolving food preferences and eating habits by making certain foods more accessible than others and portraying themselves as eating behavior models (13). As the family’s gatekeepers, parents should have adequate food literacy knowledge and skills (13). Food-illiterate parents fail to model healthy family habits, worsening when parents face financial challenges that limit their access to food (13). Two aspects of food literacy, nutritional awareness and the ability to conceptualize food, were highly connected with the quality of the family’s diet (14). Children whose parents were knowledgeable about healthy diets tend to be healthier and less overweight (14). Their diets consisted primarily of vegetables, white fish, eggs, micronutrients, and proteins, with fewer servings of meat and fewer sugar-sweetened beverages (14). Teens who said their parents helped them make healthy food choices and kept a close eye on their food choice tended to have better diets (15). Since a great deal of data on how parenting affects a child’s health and nutrition during adolescence already exists, current research focuses on parents with adolescent children. These facts suggest that improving Saudi parents’ food literacy could be a promising first step in resolving our nation’s current nutrition challenges, especially during the nutritionally vulnerable adolescent stage. Thus, to guide future interventions, this study was conducted as the first in Saudi Arabia to (1) evaluate the food literacy of Saudi parents of 10–19 years old adolescent children and (2) determine the correlates associated with parental food literacy.

## 2. Materials and methods

### 2.1. Study design and participants’ recruitment

Following a cross-sectional design and the convenience snowball sampling method, this study was conducted between 29 April and 6 June 2022. Data were collected from 1,845 Saudi parents using an online-based self-administered questionnaire. The survey link was kept open on different social media platforms (e.g., Facebook, Instagram, Twitter, Pinterest, and LinkedIn) to be filled out by eligible participants. Additionally, the author posted the survey link on public and private schools’ websites and WhatsApp groups to reach a larger sample of parents with diverse socio-demographic characteristics and age categories. Since older parents might not have internet access, the author asked their children or other family members to introduce them to the study’s aims, aiding them while responding to the survey questions when necessary. The eligibility criteria for study participants were the following: (1) being a parent for 10–19 years old adolescent children, (2) being older than 18 years old, and (3) having Saudi nationality. The age of children was decided per the World Health Organization’s (WHO) recommendations, which classify this age range (10–19 years) as the adolescent stage of life (16). To better generalize the findings, parents were recruited from almost all Saudi provinces: Riyadh, Makkah, Madinah, Eastern province, Northern borders, Jawf, Tabuk, Ha’il, Qasim, Bahah, Asir, and Najran.

### 2.2. Study variables

#### 2.2.1. Explanatory variables

Using a validated questionnaire, I collected information on the following parents’ socio-demographic characteristics: age, gender, current location, marital status (married, divorced, and widowed), number of children the parents have (sons and daughters), education level, spouses’ education level, job status (unemployed, full-time job, part-time job, and self-employed), family monthly income, the number of co-residents in the home (excluding newborns) with the number of rooms (without counting the kitchen and the bathroom) to assess the households’ crowding status. In addition, parents were asked to report any chronic disease(s) they had. Self-reported body weight and height were also collected to evaluate parents’ weight status following the WHO’s Body Mass Index (BMI) cutoff criteria (17). Based on these criteria, BMI falls into one of the following categories for persons over 20 years of age: underweight (BMI < 18.5 kg/m^2^), normal-weight (18.5–24.9 kg/m^2^), overweight (25.0–29.9 kg/m^2^), and obese (30 kg/m^2^ and above).

#### 2.2.2. Outcome variable

The outcome variable of interest for this study was the parents’ food literacy status. Thus, a valid short food literacy questionnaire (SFLQ) (18) was used to evaluate respondents’ perceived level of food literacy. As shown in Table 1, six of the 12 self-rated items asked about functional skills like understanding nutrition information and composing a balanced menu. Two items asked about interactive abilities like exchanging nutrition information with family and peers. The remaining four items inquired about critical judgment skills, such as evaluating the longer-term impact of dietary habits on health (critical FL). Respondents were given four- or five-point Likert scales with options ranging from very bad to very good, strongly disagree to strongly agree, very hard to very easy, or never to always. The questionnaire had a score ranging from 7 to 52. A higher score suggests better food literacy. The median score of 30 was used to categorize parents into two groups based on their food literacy status. A score of less than 30 indicated poor food literacy, and a score of 30 and above indicated adequate food literacy among parents in this study.

**TABLE 1:** Parental self-rated food literacy questionnaire.

Questions	Dimension	Min-Max score
1. When I have questions on nutrition, I know where I can find information on the issue.	Functional food literacy (FFL)	Disagree strongly = 1 to Agree strongly = 4; I do not have experience with these issues = 0
2. In general, how well do you understand the following types of nutritional information? (A) Nutrition information leaflets (B) Food label information (C) TV or radio programs on nutrition (D) Oral recommendations regarding nutrition from professionals. (E) Nutrition advise from family members or friends	Functional food literacy	Very bad = 1 to Very good = 5; I do not make use of this kind of information = 0
3. How familiar are you with The Healthy Food Palm (Food-based dietary guidelines), the Saudi Healthy Plate Guide, or the Healthy Food Guide for the Healthy Practitioner?	Functional food literacy	Very bad = 1 to Very good = 5
4. I know the official Saudi recommendations about fruit and vegetable consumption	Functional food literacy	Disagree strongly = 1 to Agree strongly = 4
5. I know the official Saudi recommendations about salt intake	Functional food literacy	Disagree strongly = 1 to Agree strongly = 4
6. Think about a usual day; how easy or difficult is it for you to compose a balanced meal at home?	Functional food literacy	Very hard = 1 to very easy = 4; not applicable = 0
7. In the past, how often were you able to help your family members or a friend if they had questions concerning nutritional issues?	Interactive food literacy (IFL)	1 = Never to always = 5; there have never been any questions = 0
8. There is a lot of information available on healthy nutrition today. How well do you manage to choose the information relevant to you?	Interactive food literacy (IFL)	Very bad = 1 to Very good = 5; I have not been interested in these issues = 0
9. How easy/difficult is it for you to judge if media information on nutritional issues can be trusted?	Critical food literacy (CFL)	Very difficult = 1 to very easy = 4
10. Commercials often relate foods with health. How easy/hard is it for you to judge if the presented associations are appropriate or not?	Critical food literacy (CFL)	Very hard = 1 to very easy = 4
11. How easy/hard is it for you to evaluate if a specific food is relevant for a healthy diet?	Critical food literacy (CFL)	Very hard = 1 to very easy = 4
12. How easy/hard is it for you to evaluate the longer-term impact of your dietary habits on your health?	Critical food literacy (CFL)	Very hard = 1 to very easy = 4

Although the questionnaire was based on a previously validated survey (18), it was nonetheless subjected to a face validity (or “logic validity”) evaluation to ensure its accuracy. An expert panel consisting of a public health nutritionist and four registered dietitians assessed each survey question separately. The questionnaire was double-checked to ensure it captured essential aspects of food literacy, including the ability to do basic tasks, communicate with others, and make sound judgments. A statistician [an accredited practicing dietitian (APD) and an expert on question structure] checked the survey for typical mistakes (e.g., leading, confusing, or double-barreled questions). To begin, two nutritionist researchers from Taibah University’s Department of Applied Medical Science served as pilot respondents for the study. After this, 10 parents of children aged 10–19 were recruited as a convenience sample to pilot test the survey and provide feedback on its length, clarity, and usability. This helped establish how long the average parent would take to complete the questionnaire. As a result, revisions were made to some of the existing questions to accommodate society and Saudi recommendations.

### 2.3. Ethical approval of the study protocol

The current study protocol was approved by the ethical committee of Taibah University (TU), Saudi Arabia. I adhered to the Helsinki declaration principles developed by the World Medical Association while conducting this study. Hence, all parents provided their consent to participate, verbally and electronically, before filling out the first page of the online survey. There were no risks associated with participation in this study, which was voluntary.

### 2.4. Data analysis

The collected data was exported to the Statistical Package of Social Sciences Software (SPSS) (Version 25.0. IBM Corp: Armonk, NY, USA) for analysis. A “weighting” variable was created to enhance the sample’s representation according to gender and current location. Descriptive measures, including frequency (N), percentage (%), mean, and standard deviation (SD), were obtained to summarize the study findings. The continuous variables’ normal distribution was checked using the Shapiro–Wilk test. The Mann–Whitney *U* test was used to detect mean differences between study variables composed of two groups. The chi-squared test (χ^2^) was used to identify significant associations between the explanatory variables (such as age, gender, education level, and health status) and the outcome variable of the study (food literacy). Furtherly, a multivariate analysis using the backward stepwise method of the binary logistic regression was used to point out the most significant determinants of parents’ food literacy. A *p*-value of 0.05 and below was considered significant for all analytical tests.

## 3. Results

### 3.1. Parental socio-demographic characteristics and health status

Overall, this study included a total of 1,845 Saudi parents. Of them, 56% were mothers. The overall mean age of parents was 45.1 ± 11.0. Participants were recruited from almost all Saudi provinces. More than half of the parents (57%) were overweight or obese. Most parents (84.9%) were currently married, and 65.6% had 2–3 children. Furthermore, most (64.5%) had a university education level, and 45.6% of the parents reported working full-time. Additionally, 53% of the participants made 3,000–10,000 Saudi Riyal (SR) per month (equivalent to 799–2,661 USD or 779–2,598 EUR). Roughly 39.7% of the parents’ households were either crowded or overcrowded. As for their health status, 29.3% had one or more chronic diseases (Table 2).

**TABLE 2:** Parental socio-demographic characteristics and health status.

	Overall (*N* = 1,845)		Females (*n* = 1,034)		Males (*n* = 811)		*P*-value
	Mean ± SD		Mean ± SD		Mean ± SD		
**Age in years**	45.1 ± 11.0		42.0 ± 11.0		49.0 ± 9.0		**<0**.**001**
	
	***N***	**%**	***N***	**%**	***N***	**%**	

Location							<0.001**
Riyadh	178	9.6	80	7.8	98	12.0	
Makkah	170	9.2	82	7.9	88	10.8	
Madinah	166	9.0	85	8.2	81	10.0	
Eastern province	241	13.1	55	5.3	186	22.9	
Northern borders	53	2.9	53	5.1	0	0.0	
Jawf	245	13.3	53	5.1	192	23.7	
Tabuk	130	7.0	98	9.4	32	3.9	
Ha’il	133	7.2	133	12.9	0	0.0	
Qasim	149	8.1	90	8.7	59	7.3	
Bahah	148	8.0	100	9.6	48	5.9	
Asir	126	6.8	99	8.6	27	3.4	
Najran	106	5.8	106	10.3	0	0.0	
Weight status (*n* = 1,778)^a^							<0.001**
Underweight	87	4.7	65	6.6	22	2.7	
Normal-weight	639	34.6	435	44.3	203	25.6	
Overweight	537	29.1	294	29.9	243	30.6	
Obese	515	27.9	188	19.1	327	41.1	
Marital status							<0.001**
Married	1,565	84.9	849	82.2	716	88.3	
Divorced	101	5.5	47	4.6	54	6.7	
Widowed	178	9.6	137	13.3	40	5.0	
Number of children							0.04*
One child	275	14.9	156	15.1	120	14.7	
2–3	359	19.5	222	21.4	138	17.0	
>3	1,211	65.6	656	63.5	553	68.3	
Education level							<0.001**
No formal education	72	3.9	63	6.1	8	1.0	
Elementary school level	93	5.0	37	3.6	56	6.9	
Intermediate school level	241	13.0	205	19.8	36	4.4	
Secondary school level	249	13.5	127	12.3	122	15.0	
University level	1,190	64.5	601	58.2	589	72.6	
Job status							<0.001**
Unemployed	727	39.4	565	54.6	162	20.0	
Full-time job	841	45.6	369	35.7	471	58.1	
Part-time job	118	6.4	49	4.8	68	8.4	
Self-employed	159	8.6	50	4.9	109	13.5	
Monthly income							<0.001**
None	72	3.9	52	5.0	20	2.4	
Less than 3,000 SR (<799 USD$/779 euro €)	82	4.5	38	3.6	44	5.5	
3,000–10,000 SR (equivalent to 799–2,661 USD$/779–2,598 euro €)	977	53.0	597	57.8	380	46.9	
10,000–25,000 SR (equivalent to 2,661–6,652 USD $/2,597–6,497 euro €)	570	30.9	270	26.1	301	37.1	
More than 25,000 SR (>6,652 USD$/6,497 euro €)	143	7.8					
Household crowding index							<0.001**
≤1^b^	1,113	60.3	683	66.1	429	53.0	
>1^c^	468	25.4	172	16.7	295	36.5	
>1.5^d^	264	14.3	178	17.2	86	10.6	
**Prevalence of chronic diseases (*n* = 540; among diseased parents)**							
Cardiovascular diseases (CVD)	25	3.2	7	1.9	18	4.3	0.04*
Diabetes	217	28.1	62	17.3	155	37.4	<0.001**
Hypertension	217	28.0	65	18.1	152	36.7	<0.001**
Chronic kidney diseases (CKD)	6	0.8	2	0.6	4	1.0	0.26
Liver diseases	23	3.0	21	5.9	2	0.5	0.001*
Osteoporosis	8	1.0	7	1.9	1	0.2	0.07
Cancer	11	1.4	1	0.3	9	2.2	0.003*
Respiratory diseases	79	10.2	23	6.4	56	13.5	<0.001**
Anemia	130	16.8	114	31.8	16	3.9	<0.001**
Others	57	7.4	56	15.6	1	0.2	<0.001**

^a^Some parents did not report their body weight, and this is why some data were missing; ^*b*^no crowding; ^c^crowded; ^d^over-crowded; *significant at *p*-value < 0.05 for χ^2^ test; **significant at *p*-value < 0.001 for χ^2^ test; bold value was significant based on the Mann–Whitney *U* test.

### 3.2. Parental food literacy status and its correlates

Nearly half the parents (46%) had poor food literacy scores. The correlates associated with parental food literacy are shown in Table 3. The mean age of food-illiterate parents (46 ± 10) was higher than those with adequate food literacy levels (45 ± 12), *p* = 0.02. Father participants (58.6%) were more food illiterate than mothers (36.1%), *p* < 0.001. More than half the participants residing in Riyadh (54.8%), Northern borders (100%), Jawf (78.3%), and Ha’il (60%) had poor food literacy scores, *p* < 0.001. The majority of obese parents (66.6%) had poor food literacy scores, exceeding that reported among parents with different weight statuses, particularly underweight (24%), *p* < 0.001.

**TABLE 3:** The correlates associated with parental food literacy.

Correlates	Parental food literacy				
	Adequate (*N* = 997)		Poor (*N* = 848)		*P*-value
	*N*	%	*N*	%	
Gender					<0.001**
Female	661	63.9	373	36.1	
Male	336	41.4	475	58.6	
Location					<0.001**
Riyadh	80	45.2	98	54.8	
Makkah	105	62.0	65	33.0	
Madinah	111	67.0	55	33.0	
Eastern province	139	57.5	102	42.5	
Northern borders	0	0.0	53	100.0	
Jawf	53	21.7	192	78.3	
Tabuk	86	66.7	43	33.3	
Ha’il	53	40.0	80	60.0	
Qasim	79	52.7	70	47.3	
Bahah	90	61.3	57	38.7	
Asir	111	88.0	15	12.0	
Najran	89	83.3	18	16.7	
Weight status (*n* = 1,778)^a^					<0.001**
Underweight	66	76.0	21	24.0	
Normal-weight	334	52.4	304	47.6	
Overweight	284	52.9	253	47.1	
Obese	172	33.4	343	66.6	
Marital status					0.21
Married	838	53.5	728	46.5	
Divorced	52	51.6	49	48.4	
Widowed	107	60.0	71	40.0	
Number of children					0.42
One child	158	57.5	117	42.5	
2–3	188	52.3	171	47.7	
>3	650	53.7	560	46.3	
Education level					<0.001**
Noformal education	54	75.9	17	24.1	
School education level	333	57.1	250	42.9	
University education level	610	51.2	580	48.8	
Job status					<0.001**
Not employed	442	60.8	285	39.2	
Employed (full-time; part-time; and self-employed)	554	49.6	563	50.4	
Monthly income					0.002*
None or less than 3,000 SR	77	50.1	77	49.9	
3,000–25,000 SR	822	53.1	726	46.9	
More than 25,000 SR	98	68.2	46	31.8	
Prevalence of chronic disease(s)					<0.001**
No	630	48.3	674	51.7	
Yes	366	67.8	174	32.2	
Household crowding status					<0.001**
Not crowded	707	63.6	406	36.4	
Crowded	289	39.5	442	60.5	

^a^Some parents did not report their body weight and this is why some data were missing [underweight parents *n* = 87 (66 were food literate and *n* = 21 had poor food literacy); normal-weight parents *n* = 638 (334 were food literate and *n* = 304 had poor food literacy); overweight parents *n* = 537 (284 were food literate and *n* = 253 had poor food literacy); and obese parents *n* = 515 (172 were food literate and 343 had poor food literacy)].

*Significant at *p*-value < 0.05 for χ^2^ test; **significant at *p*-value < 0.001 for χ^2^ test.

Also, 48.8% of parents with a university education level were food illiterate compared to those who reported a lower education level [school education (42.9%) or having no formal education (24.1%)], *p* < 0.001. Around half (50.4%) of employed parents (full-time, part-time, and self-employed) had poor food literacy compared to unemployed parents (39.2%), *p* < 0.001. Roughly 49.9% of parents who reported having no income or lower than 3,000 SR monthly income had poor food literacy scores, *p* = 0.002. The prevalence of food illiteracy was highest among healthy parents (51.7%), as opposed to 32.2% of parents having one or more chronic diseases. Parents living in crowded households had higher levels of food illiteracy than those who were not (60.5 vs. 36.4%, *p* 0.001). Parental marital status and the number of children the parents have shown no significant associations with parental food literacy (*p* = 0.21 and *p* = 0.42, respectively) (Table 3).

### 3.3. The determinants of parental food literacy: Binary logistic regression analysis

Table 4 demonstrates that fathers were 2.4 times more likely than mothers to be food illiterate (OR = 2.4, CI = 1.9–3.0, *p* < 0.001). Parents residing in Riyadh, Northern borders, Jawf, or Ha’il provinces had a risk of poor food literacy that was three times higher than those living in other Saudi provinces (OR = 3.2, CI = 2.6–3.9, *p* < 0.001). Obese/overweight parents had a 60% higher risk of being food illiterate (vs. underweight parents OR = 1.6, CI = 1.3–2.1, *p* < 0.001). Healthy parents (reference), compared to those having one or more chronic diseases, had a 60.0% higher probability of having poor food literacy (OR = 0.4, CI = 0.3–0.6, *p* < 0.001).

**TABLE 4:** The determinants of parental food literacy: Binary logistic regression analysis.

Dependent variable: Food literacy [adequate (reference) vs. poor]	AOR (95% CI)	*P*-value
**Gender** (Reference: Female)	—	—
Male	2.4 (1.9–3.0)	<0.001**
**Current location** (Residence: Other location)		
Riyadh; Northern borders; Jawf; Ha’il	3.2 (2.6–3.9)	<0.001**
**Weight status** (Reference: Underweight)	—	—
Overweight or obese	1.6 (1.3–2.1)	<0.001**
**Health status** (Reference: having no chronic diseases)	—	—
Having chronic disease(s)	0.4 (0.3–0.6)	<0.001**
**Education level** (Reference: no formal education)	—	—
Educated (school/university)	3.0 (1.6–5.8)	0.001*
**Family monthly income** (Reference: None or less 3,000 SR)	—	—
3,000–25,000 SR	0.6 (0.4–0.9)	0.02*
More than 25,000 SR	0.3 (0.2–0.6)	<0.001**
**Household crowding status** (Reference: Not crowded)		
Crowded	1.9 (1.5–2.4)	<0.001**

Variables entered in step 1: age, gender, residence, weight status, education level, job status, monthly income, prevalence of chronic disease, and households’ crowding status; AOR, adjusted odds ratio; CI, confidence interval; *significant at *p*-value < 0.05; **significant at *p*-value < 0.001.

Added to these, educated parents (with school or university education levels) had triple the risk of being food illiterate compared to their counterparts who have no formal education (OR = 3, CI = 1.6–5.8, *p* < 0.001). Parents making less than 3,000 Saudi Riyal (SR) per month were 40% more likely to be food illiterate than those who reported 3,000–25,000 SR (OR = 0.6, CI = 0.4–0.9, *p* = 0.02), and 70.0% more likely to be food illiterate than those making more than 25,000 SR (OR = 0.3, CI = 0.2–0.6, *p* < 0.001). Parents living in crowded households had doubled the risk of food illiteracy compared to those who were not (OR = 1.9, CI = 1.5–2.4, *p* < 0.001) (Table 4).

## 4. Discussion

A person’s level of food literacy is a significant factor in determining how well they can maintain a healthy nutrition status (5). Consequentially, academics and various stakeholders in the healthcare industry increasingly regard nutritional literacy as an essential component in health promotion (19). To the best of the author’s knowledge, this population-based, cross-sectional study is the first to evaluate parents’ food literacy and the factors that influence it in Saudi Arabia. The study respondents included 1,845 parents (the mean age was 45.1 ± 11.0, and 56% were mothers); I found that nearly half (46%) had poor food literacy scores. The binary logistic regression analysis showed that parental gender, residence, weight status, health status, job status, education level, monthly income, and household crowding status were the most significant factors contributing to the determination of parental food literacy in this study.

### 4.1. Parental food literacy and potential intervention programs

According to the survey results, nearly half of the parents (46%) had low levels of food literacy. This result is consistent with a recent literature review on testing food literacy in the Middle East and North Africa (MENA) area, which found that most people in these countries had inadequate food and/or nutrition literacy, particularly in the domain of skills rather than cognition (6). It appears that the current food literacy status of Saudi parents is worse than that observed in Canada, where most parents had good food literacy competencies, manifested by selecting foods based on nutrition labels (81%), planning meals before going to the market (91%) using grocery lists (95%), cooking with whole and basic ingredients (66.6%), and having advanced cooking skills (66.6%) (20). Like food literacy, nutrition literacy is “the degree to which people can obtain, process, and understand basic diet information and the tools needed to make appropriate nutrition decisions” (21). One recent study showed that most Greek parents were nutritionally literate, which was shown to positively influence the feeding patterns of their children (22). Consistent with the present study’s findings, one study showed that parents experienced various unpleasant emotions when feeding their children, including anxiety, difficulty, and concern (23). Furthermore, several challenges were encountered by parents when trying to share food literacy skills with their children, including time pressure, safety concerns, lack of interest from children, and conflict between siblings (24). The influence of parents was further demonstrated in a study showing that children reported a higher healthy eating index if their parents had better nutrition literacy (25).

A recent review enlightened the devaluation of food literacy and nutrition literacy topics in MENA countries (6). It found that countries outside MENA boundaries had better food and nutrition literacy status than those within in terms of assessment and food literacy programming (6). Investing in nutrition programs and instructional techniques is necessary to raise a community’s food literacy (6). Thus, the current findings emphasize the necessity of provoking serious steps to carry out food literacy interventions parallel to those developed and proven successful in other countries. The OzHarvest’s Nutrition Education and Skills Training (NEST) Program has been designed and implemented in Australia to improve adults’ food literacy (26). The OzHarvest’s NEST Program is a 6-week, 15-h guided public health nutrition program, integrating a series of nutrition activities, goal setting, and practical cooking lessons, utilizing recipes from OzHarvest’s Everyday Cookbook and culminating in the sharing of a meal together (26). In the post-intervention period, participants were shown to experience improvements in food security status, cooking confidence, food preparation behaviors, and nutrition knowledge (26). Free of charge, the Food Sensations for Adults (FSA) program is designed to teach people from lower- and middle-income backgrounds about healthy eating and how to shop for and cook nutritious meals (27). Every session is an opportunity to learn something new in a relaxed and supportive setting, and participants are encouraged to try new techniques and foods (27). The FSA program also promoted improved food literacy in 61–74% of adult program participants (low and middle-income Australians), manifested by increased self-reported fruit and vegetable intake, planning, management, selection, and food preparation (27). Similarly, the Food Sensation for Parents (FSP) program assisted parents of children up to 5 years old in promoting healthy family eating habits, introducing solid foods to their children, managing mealtimes and lunchboxes, reading food labels, practicing food safety precautions, budgeting, and meal planning. (28). Parents need to be aware of the worth of parent-child cooking activities, which have been proven to lessen dietary concerns in children, such as picky eating and playing with food and utensils during meals (29). Moreover, children who participated in culinary activities had more varied diets, including more fish, soy products, vegetables, and milk, according to a study based on data from Japan’s national nutrition survey on preschool children, a survey of families across the country with toddlers and preschoolers. (29). The importance of parents gaining self-confidence in the kitchen when cooking was highlighted in another cross-sectional study with 657 child-parent pairs from São Paulo, Brazil. This was connected with the children eating less highly processed food (30).

The Kingdom of Saudi Arabia should start considering strategies for improving parents’ food literacy, including knowledge of buying groceries, reading nutrition labels, integrating them into nutrition education lessons with their children in school settings, and how to increase their confidence while cooking. Schools are ideal for concurrently educating parents and their children about nutrition (31). School activities might include homework assignments for parents, sending home nutrition educational materials, inviting parents to attend nutrition classes, and inviting parents to present in special events such as School Lunch Week or tasting events (31). Nonetheless, formal evaluation and monitoring of the existing food literacy programs must be conducted to determine the degree of implementation and whether they are appropriate in the Saudi Arabian context.

### 4.2. Parental involvement in adolescents’ eating habits

Children develop their eating habits primarily from their parents, both genetically and in terms of the setting in which they are raised. One study showed that adolescents’ diet quality scores positively correlate with their perceptions of their parents’ attitudes toward nutrition, their peers’ dietary habits, and caregivers’ assessments of parental surveillance of adolescent dietary behavior (32). They reported that parental support and close food choice monitoring had resulted in higher-quality diets (32). Meanwhile, 20.5% of adolescents reported a lack of parental rules on eating, such as breakfast not being mandatory, meals in front of the TV being allowed, and not restricting sweets and soft drinks, had resulted in lower-quality diets (32). Teenagers who did not adhere to their parents’ dietary guidelines were more likely to miss breakfast, consume fewer fruits and vegetables, and consume sweets, soft drinks, and energy drinks often (33). Trends like an increase in eating occasions, portion sizes, energy density, snacking frequency, skipping breakfast, and eating meals away from home can negatively affect the diet quality and energy balance of children and adolescents. (33). Intriguingly, it has been established that helping with family meal preparation improves adolescents’ food quality and eating behaviors (33). Programs or interventions that teach cooking skills to adolescents could be good initiatives to improve their nutrition status; of these programs are OzHarvest’s primary-school Food Education and Sustainability Training (FEAST) program (34), Food Sensation for Schools (FSS) (35), Fuel Your Future (FYF) (36), Teens CAN: Comprehensive Food Literacy in Cooking, Agriculture, and Nutrition (37), Cook IT UP (38), and Food Literacy Project’s Youth Community Agriculture Program (YCAP) (39). Ensuring adequate parental food literacy could positively impact everyone in the community by improving the health and nutritional status of the parents and their children.

### 4.3. The correlates of parental food literacy

A person’s ability to maintain a healthy diet is influenced by various factors, including socioeconomic status, cultural norms, and level of nutritional literacy (40). This study’s findings reveal that parental gender, residence, weight status, health status, job status, education level, monthly family income, and household crowding status contributed most significantly to determining parents’ food literacy status. They align with the results of other research that assessed gender, income, health, and weight status in the adult population (21, 41, 42). Similar conclusions were drawn from studies involving children and adolescents, which also discovered correlations between the participants’ socio-demographic characteristics and food literacy (43, 44). The current study showed that fathers were 2.4 times more likely than mothers to be food illiterate. This finding is warrantable and not unpredicted, as in Arab cultures, mothers are more involved in cooking and shopping activities than fathers. In addition, the literature showed that up to 50% of gender differences in food preferences might be explained by health attitudes and motivations to lose weight (45). This could account for the current study’s higher level of food literacy among women. Additionally, these findings corroborated data from a recent study showing that women paid more attention to nutritional properties than men and obtained higher food literacy scores (46).

Parents who are overweight or obese showed an increased risk of being food illiterate by 60%. This conclusion could be explained by the fact that people with low food literacy are more likely to engage in high-risk eating behaviors that lead to weight gain. Risky eating habits contributing to weight gain include a diet high in fast food, skipping meals, and consuming a disproportionate number of processed foods (47). Compared to those with chronic disease(s), healthy participants had a 60.0% higher probability of expressing poor food literacy in the current study; this link may be due to parents dealing with chronic disorders needing to consult doctors and dietitians more often. Additionally, they may be more interested in learning about nutrition and food from various sources, such as the nutritional value of foods, how to cook a healthy meal, and how nutrition may be utilized to control non-communicable diseases (42).

The current study indicated an unexpectedly high probability of food illiteracy among educated parents. This result was unusual, but it is reasonable given that various confounding factors influence parental food literacy in addition to education level (6). Habits, label reading, consumption patterns, academic success, availability of healthy foods, variety in the diet, and nutrient sufficiency were all potential confounding variables (6). Despite the scarcity of research in this field, the findings of this study were comparable with those of a Turkish study, which revealed that participants’ food literacy scores decreased in proportion to their academic ranks (42). These results might be explained by knowing that educated individuals are more likely to be employed, leading to a busy lifestyle in which many people struggle to find the time to make nutritious meals and pay attention to nutritional information (48). Busy family lives were connected with a greater likelihood of parents giving their children fast food (48). Previous research suggested that parents appeared to be aware of the need to feed their children nutritious meals and snacks. Still, parents were concerned they would become distracted throughout the day owing to their demanding work schedules, leading them to drive by fast food restaurants to feed their children for a quick meal (49). However, more research is needed to investigate these findings thoroughly.

When the food literacy scores of the participants were assessed according to their residential regions, it was discovered that parents from the provinces of Riyadh, Northern borders, Jawf, and Ha’il had a threefold risk of being food illiterate compared with parents from other provinces. Since this is the first study of its kind in Saudi Arabia, more research is needed to understand the results fully. This is especially true given the impossibility of initiating a comprehensive description of the areas where participants had a good level of food literacy. While some of these regions are rural, others are metropolitan and central. However, this finding may help target future food literacy programs to prioritize vulnerable populations for intervention in Saudi Arabia. Lower monthly income and crowded households also predicted worse food literacy scores among parents. This might be related to food illiteracy being frequently associated with a household’s food security status (27). Food-insecure people, especially those with low monthly incomes, do not usually focus on their diet quality (a utility pillar of food security). Food insecurity and food literacy thus have a dual relationship (27).

### 4.4. Limits and strengths

The study’s cross-sectional design allows for drawing associations but not causal relationships. Information bias and misreporting could be assumed as the questionnaire was online-based and self-administered. Furthermore, this study did not adequately present households lacking internet access, and data on the households’ diet quality were not collected. On the other hand, public health improvements necessitate research on essential elements, such as the level of food literacy in healthy eating. Therefore, despite the above limitations, the findings of this study are expected to fill research gaps in food literacy and contribute to existing scholarship in this area. In addition, this is the first study of its kind to examine the relationship between parental food literacy and other variables in Saudi Arabia. The large and diverse sample also allows us to acquire a more accurate depiction of the level of food literacy among parents in the kingdom of Saudi Arabia. The sample’s representativeness of the relevant population facilitates a more realistic view of parents’ food literacy in Saudi Arabia.

## 5. Conclusion and future perspectives

This study is the first to provide data on food literacy status among Saudi parents. Its findings reveal that nearly half of the Saudi parents sampled had poor food literacy. Parental gender, residence, weight status, health status, job status, education level, monthly income, and household crowding status contributed most significantly to predicting parental food literacy. Parents are the essential first teachers for their children; therefore, food illiteracy could negatively affect the nutritional outcomes of the whole family. The current study findings should be employed in future programming and policy-making approaches to reach Saudi parents for necessary food literacy interventions. These interventions could bolster their confidence in cooking and knowledge of how to buy groceries, read nutrition labels, and provide nutrition education lessons for their children. Intervention suggestions are context-specific and should be evaluated for their applicability and effectiveness, considering the nutrition vulnerabilities of every population group. In closing, programs to address food literacy in parents are indispensable in Saudi Arabia, and policymakers should prioritize them in their agendas.

## Data availability statement

The datasets presented in this study are not readily available due to confidentiality reasons, requests to access the data can be directed to the author KB, kbookari@taibahu.edu.sa.

## Ethics statement

The studies involving human participants were reviewed and approved by Taibah University, Saudi Arabia. The patients/participants provided their written informed consent to participate in this study.

## Author contributions

KB conceived the study, conducted the analyses, interpreted the results, wrote the manuscript, critically reviewed the manuscript, and approved the final version submitted for publication.
